# (*E*)-2-(1,1-Di­cyclo­hexyl-3-phenyl­all­yl)-5,5-dimethyl-1,3,2-dioxaborinane

**DOI:** 10.1107/S1600536813021739

**Published:** 2013-08-10

**Authors:** Gamal A. El-Hiti, Keith Smith, Mark C. Elliott, Dyfyr Heulyn Jones, Benson M. Kariuki

**Affiliations:** a Department of Optometry, College of Applied Medical Sciences, King Saud University, PO Box 10219, Riyadh 11433, Saudi Arabia; b School of Chemistry, Cardiff University, Main Building, Park Place, Cardiff CF10 3AT, Wales

## Abstract

The crystal structure of the title compound, C_26_H_39_BO_2_, which contains no strong hydrogen bond donors, displays only long C—H⋯O contacts between inversion-related pairs of mol­ecules. The structure contains layers rich in oxygen and boron parallel to the *ac* plane. The dioxaborinane ring adopts an envelope conformation with the C atom attached to the two methyl groups as the flap .

## Related literature

For the synthesis and applications of allyl­boronic esters, see: Lombardo *et al.* (2002 ▶); Carosi & Hall (2007 ▶); Althaus *et al.* (2010 ▶); Fandrick *et al.* (2010 ▶); Clary *et al.* (2011 ▶); Hesse *et al.* (2012 ▶); Incerti-Pradillos *et al.* (2013 ▶). For the X-ray structure of a boronic ester, see: Sopková-de Oliveira Santos *et al.* (2003 ▶).
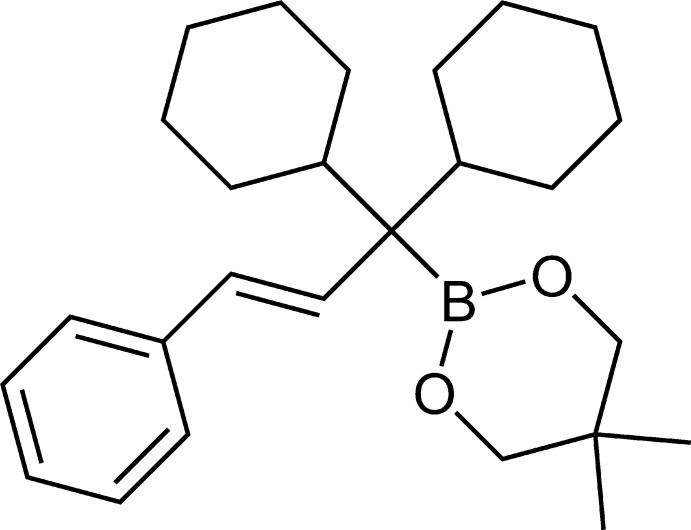


## Experimental

### 

#### Crystal data

C_26_H_39_BO_2_
*M* *_r_* = 394.38
Triclinic, 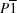

*a* = 9.4967 (3) Å
*b* = 11.2837 (2) Å
*c* = 12.0297 (4) Å
α = 109.897 (2)°
β = 96.388 (2)°
γ = 102.048 (2)°
*V* = 1161.90 (6) Å^3^
*Z* = 2
Mo *K*α radiation
μ = 0.07 mm^−1^
*T* = 150 K
0.38 × 0.30 × 0.28 mm

#### Data collection

Nonius KappaCCD diffractometer
Absorption correction: multi-scan (*DENZO*/*SCALEPACK*; Otwinowski & Minor, 1997 ▶) *T*_min_ = 0.975, *T*_max_ = 0.981
8277 measured reflections
5287 independent reflections
4303 reflections with *I* > 2σ(*I*)
*R*_int_ = 0.027

#### Refinement

*R*[*F* ^2^ > 2σ(*F* ^2^)] = 0.050
*wR*(*F* ^2^) = 0.128
*S* = 1.03
5287 reflections
264 parameters
H-atom parameters constrained
Δρ_max_ = 0.32 e Å^−3^
Δρ_min_ = −0.20 e Å^−3^

### 

Data collection: *COLLECT* (Nonius, 2000 ▶); cell refinement: *SCALEPACK* (Otwinowski & Minor, 1997 ▶); data reduction: *DENZO* (Otwinowski & Minor, 1997 ▶) and *SCALEPACK*; program(s) used to solve structure: *SIR92* (Altomare *et al.*, 1994) ▶; program(s) used to refine structure: *SHELXL97* (Sheldrick, 2008 ▶); molecular graphics: *ORTEP99* for Windows (Farrugia, 2012 ▶); software used to prepare material for publication: *WinGX* (Farrugia, 2012 ▶) and *CHEMDRAW Ultra* (Cambridge Soft, 2001 ▶).

## Supplementary Material

Crystal structure: contains datablock(s) I, New_Global_Publ_Block. DOI: 10.1107/S1600536813021739/go2094sup1.cif

Structure factors: contains datablock(s) I. DOI: 10.1107/S1600536813021739/go2094Isup2.hkl

Supplementary material file. DOI: 10.1107/S1600536813021739/go2094Isup3.cml

Additional supplementary materials:  crystallographic information; 3D view; checkCIF report

## Figures and Tables

**Table 1:** Hydrogen-bond geometry (Å, °)

*D*—H⋯*A*	*D*—H	H⋯*A*	*D*⋯*A*	*D*—H⋯*A*
C22—H22*B*⋯O2^i^	0.99	2.69	3.5773 (18)	150

Symmetry code: (i) 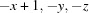.

**Fig. 1.**
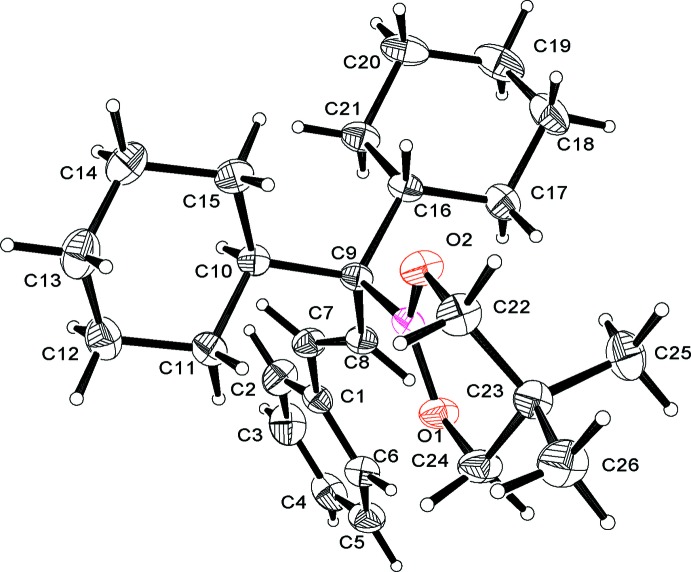
A molecule showing atom labels and 50% probability displacement ellipsoids for non-H atoms.

**Fig. 2.**
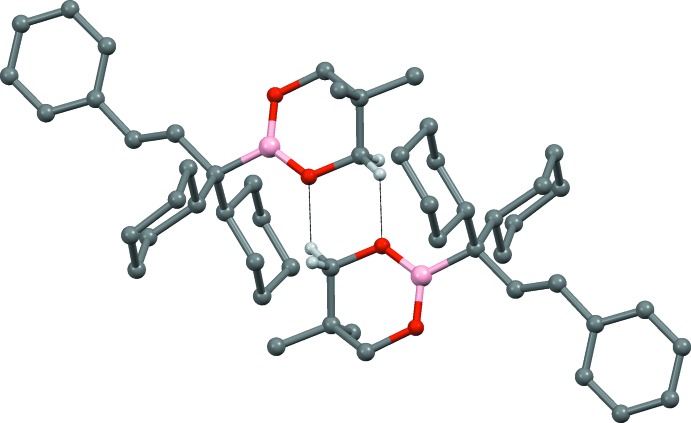
A pair of molecules showing C—H···O interactions as dotted lines.

**Fig. 3.**
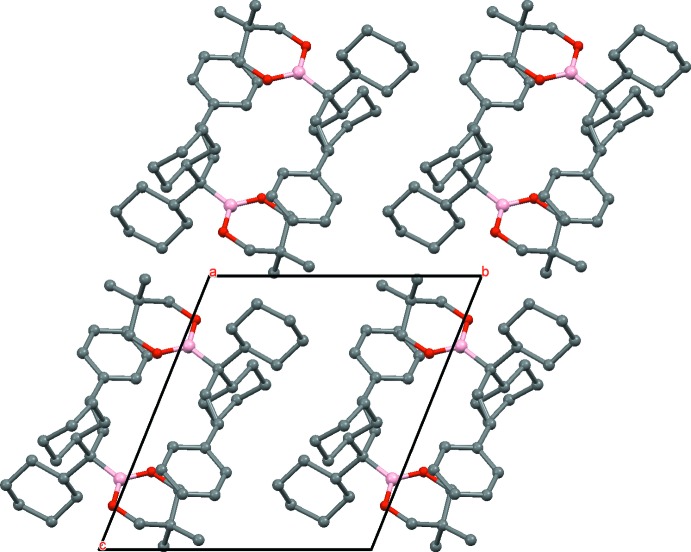
Molecular packing in the crystal structure showing oxygen and boron rich layers.

## supplementary crystallographic information

### 1. Comment

The title compound I, a useful synthetic intermediate, was synthesized by the reaction of *(E)*-dicyclohexylstyrylborane with the anion of dichloromethyl methyl ether followed by esterification with 2,2-dimethyl-1,3-propanediol. Allylboronic esters have been synthesized from the reaction of lithiated carbamates with vinylboranes [Althaus *et al.* (2010)], and from the reaction of primary allyl halides with pinacolborane and magnesium [Incerti-Pradillos *et al.* (2013), Clary *et al.* (2011)]. Allylboronic esters are important synthetic intermediates, which have been shown to react with aldehydes to give homoallylic alcohols [Lombardo *et al.* (2002)], with the control of the newly-generated stereogenic centre possible through use of a chiral catalyst [Carosi *et al.* (2007)]. Allylboronic esters take part in a zinc alkoxide catalysed reaction with ketones to give the corresponding homoallylic alcohol products [Fandrick *et al.* (2010)], and also take part in a proto-deboronation reaction which has been used to synthesize the pheromone of the Californian red scale beetle [Hesse *et al.* (2012)]. For the X-ray structure of a boronic ester, see: Sopková-de Oliveira Santos *et al.* (2003).

In the molecule (Figure 1), the two cyclohexyl groups assume a chair conformation and an envelope conformation is observed for the dioxaborinane ring. The phenylallyl group is not planar as the plane through the double bond makes an angle of 20.84 ° with the phenyl group. There are no strong hydrogen bond donors in the structure. Long contacts of C—H···O type occur between pairs of molecules to form loosely bound dimers (Figure 2). The dimers are stacked along the *a*-axis to form a structure with layers rich in oxygen and boron parallel to the *ac* plane (Figure 3).

### 2. Refinement

H atoms were positioned geometrically and refined using a riding model with *U*_iso_(H) = 1.2 times *U*_eq_ for the atom they are bonded to except for the methyl groups where 1.5 times *U*_eq_ was used with free rotation about the C—C bond. Of the low angle reflections not included in the refinement only (0 0 1) and (0 1 0) were omitted due to low intensities consistent with being obscured by the beamstop. The rest were eliminated automatically during data processing possibly as overloads.

### Crystal data

**Table:**

C_26_H_39_BO_2_	*Z* = 2
*M**_r_* = 394.38	*F*(000) = 432
Triclinic, *P*1	*D*_x_ = 1.127 Mg m^−^^3^
Hall symbol: -P 1	Mo *K*α radiation, λ = 0.71073 Å
*a* = 9.4967 (3) Å	Cell parameters from 4303 reflections
*b* = 11.2837 (2) Å	θ = 2.2–27.5°
*c* = 12.0297 (4) Å	µ = 0.07 mm^−^^1^
α = 109.897 (2)°	*T* = 150 K
β = 96.388 (2)°	Block, colourless
γ = 102.048 (2)°	0.38 × 0.30 × 0.28 mm
*V* = 1161.90 (6) Å^3^	

### Data collection

**Table:**

Nonius KappaCCD diffractometer	5287 independent reflections
Radiation source: fine-focus sealed tube	4303 reflections with *I* > 2σ(*I*)
Graphite monochromator	*R*_int_ = 0.027
CCD slices, ω and phi scans	θ_max_ = 27.5°, θ_min_ = 2.1°
Absorption correction: multi-scan (*DENZO*/*SCALEPACK*; Otwinowski & Minor, 1997)	*h* = −12→12
*T*_min_ = 0.975, *T*_max_ = 0.981	*k* = −14→14
8277 measured reflections	*l* = −15→12

### Refinement

**Table:**

Refinement on *F*^2^	Primary atom site location: structure-invariant direct methods
Least-squares matrix: full	Secondary atom site location: difference Fourier map
*R*[*F*^2^ > 2σ(*F*^2^)] = 0.050	Hydrogen site location: inferred from neighbouring sites
*wR*(*F*^2^) = 0.128	H-atom parameters constrained
*S* = 1.03	*w* = 1/[σ^2^(*F*_o_^2^) + (0.0488*P*)^2^ + 0.4651*P*] where *P* = (*F*_o_^2^ + 2*F*_c_^2^)/3
5287 reflections	(Δ/σ)_max_ = 0.004
264 parameters	Δρ_max_ = 0.32 e Å^−^^3^
0 restraints	Δρ_min_ = −0.20 e Å^−^^3^

### Special details

**Table:**

Geometry. All s.u.'s (except the s.u. in the dihedral angle between two l.s. planes) are estimated using the full covariance matrix. The cell s.u.'s are taken into account individually in the estimation of s.u.'s in distances, angles and torsion angles; correlations between s.u.'s in cell parameters are only used when they are defined by crystal symmetry. An approximate (isotropic) treatment of cell s.u.'s is used for estimating s.u.'s involving l.s. planes.
Refinement. Refinement of *F*^2^ against ALL reflections. The weighted *R*-factor *wR* and goodness of fit *S* are based on *F*^2^, conventional *R*-factors *R* are based on *F*, with *F* set to zero for negative *F*^2^. The threshold expression of *F*^2^ > 2σ(*F*^2^) is used only for calculating *R*-factors(gt) *etc*. and is not relevant to the choice of reflections for refinement. *R*-factors based on *F*^2^ are statistically about twice as large as those based on *F*, and *R*- factors based on ALL data will be even larger.

### Fractional atomic coordinates and isotropic or equivalent isotropic displacement parameters (Å^2^)

**Table:**

	*x*	*y*	*z*	*U*_iso_*/*U*_eq_	
C1	0.24096 (15)	0.23892 (13)	0.62817 (11)	0.0235 (3)	
C2	0.22913 (18)	0.35166 (15)	0.71836 (13)	0.0348 (3)	
H2	0.2828	0.4349	0.7232	0.042*	
C3	0.14007 (19)	0.34351 (17)	0.80085 (14)	0.0411 (4)	
H3	0.1319	0.4211	0.8607	0.049*	
C4	0.06330 (17)	0.22337 (17)	0.79648 (14)	0.0382 (4)	
H4	0.0029	0.2179	0.8534	0.046*	
C5	0.07497 (17)	0.11101 (16)	0.70869 (14)	0.0348 (3)	
H5	0.0228	0.0280	0.7055	0.042*	
C6	0.16250 (16)	0.11876 (13)	0.62506 (13)	0.0281 (3)	
H6	0.1689	0.0407	0.5648	0.034*	
C7	0.33581 (15)	0.25145 (13)	0.54118 (12)	0.0255 (3)	
H7	0.4098	0.3312	0.5644	0.031*	
C8	0.32693 (14)	0.16134 (12)	0.43362 (11)	0.0221 (3)	
H8	0.2530	0.0818	0.4117	0.027*	
C9	0.42193 (14)	0.17080 (12)	0.34118 (11)	0.0205 (3)	
C10	0.58492 (14)	0.24810 (12)	0.40612 (11)	0.0218 (3)	
H10	0.5813	0.3293	0.4725	0.026*	
C11	0.65750 (15)	0.16852 (13)	0.46484 (12)	0.0260 (3)	
H11A	0.5978	0.1466	0.5210	0.031*	
H11B	0.6589	0.0856	0.4014	0.031*	
C12	0.81407 (16)	0.24166 (14)	0.53369 (13)	0.0330 (3)	
H12A	0.8121	0.3195	0.6031	0.040*	
H12B	0.8576	0.1844	0.5655	0.040*	
C13	0.90896 (17)	0.28434 (15)	0.45299 (15)	0.0380 (4)	
H13A	0.9200	0.2064	0.3883	0.046*	
H13B	1.0079	0.3364	0.5010	0.046*	
C14	0.83863 (17)	0.36592 (15)	0.39717 (15)	0.0361 (4)	
H14A	0.8996	0.3908	0.3430	0.043*	
H14B	0.8349	0.4471	0.4618	0.043*	
C15	0.68323 (15)	0.29066 (13)	0.32573 (13)	0.0283 (3)	
H15A	0.6875	0.2125	0.2577	0.034*	
H15B	0.6400	0.3466	0.2918	0.034*	
C16	0.35441 (15)	0.23557 (12)	0.25946 (12)	0.0229 (3)	
H16	0.4152	0.2331	0.1963	0.027*	
C17	0.19670 (16)	0.16006 (14)	0.19300 (13)	0.0309 (3)	
H17A	0.1928	0.0671	0.1500	0.037*	
H17B	0.1315	0.1645	0.2522	0.037*	
C18	0.14178 (19)	0.21631 (17)	0.10238 (15)	0.0404 (4)	
H18A	0.2011	0.2042	0.0387	0.048*	
H18B	0.0384	0.1683	0.0635	0.048*	
C19	0.1523 (2)	0.36161 (18)	0.16395 (16)	0.0436 (4)	
H19A	0.1255	0.3970	0.1021	0.052*	
H19B	0.0814	0.3726	0.2187	0.052*	
C20	0.30661 (19)	0.43826 (15)	0.23583 (14)	0.0364 (4)	
H20A	0.3073	0.5301	0.2804	0.044*	
H20B	0.3754	0.4379	0.1797	0.044*	
C21	0.35828 (17)	0.37906 (13)	0.32525 (13)	0.0287 (3)	
H21A	0.4597	0.4290	0.3689	0.034*	
H21B	0.2940	0.3855	0.3852	0.034*	
C22	0.51831 (16)	−0.10974 (13)	0.09454 (13)	0.0290 (3)	
H22A	0.6131	−0.1146	0.1338	0.035*	
H22B	0.5259	−0.1158	0.0115	0.035*	
C23	0.39668 (15)	−0.22485 (12)	0.08888 (12)	0.0256 (3)	
C24	0.37886 (18)	−0.20513 (13)	0.21769 (12)	0.0301 (3)	
H24A	0.2915	−0.2720	0.2158	0.036*	
H24B	0.4659	−0.2183	0.2607	0.036*	
C25	0.25412 (17)	−0.23168 (15)	0.01216 (14)	0.0363 (4)	
H25A	0.1768	−0.3060	0.0092	0.055*	
H25B	0.2688	−0.2427	−0.0697	0.055*	
H25C	0.2250	−0.1507	0.0477	0.055*	
C26	0.44291 (19)	−0.35104 (14)	0.03502 (14)	0.0373 (4)	
H26A	0.5375	−0.3438	0.0822	0.056*	
H26B	0.4524	−0.3656	−0.0486	0.056*	
H26C	0.3685	−0.4246	0.0369	0.056*	
B1	0.42494 (16)	0.02685 (14)	0.25703 (13)	0.0210 (3)	
O1	0.36243 (11)	−0.07729 (8)	0.28350 (8)	0.0261 (2)	
O2	0.49222 (11)	0.01427 (9)	0.16035 (8)	0.0273 (2)	

### Atomic displacement parameters (Å^2^)

**Table:**

	*U*^11^	*U*^22^	*U*^33^	*U*^12^	*U*^13^	*U*^23^
C1	0.0250 (7)	0.0294 (6)	0.0191 (6)	0.0096 (5)	0.0060 (5)	0.0110 (5)
C2	0.0404 (9)	0.0318 (7)	0.0276 (7)	0.0065 (6)	0.0118 (6)	0.0056 (6)
C3	0.0444 (9)	0.0484 (9)	0.0257 (8)	0.0151 (8)	0.0147 (7)	0.0040 (7)
C4	0.0306 (8)	0.0644 (10)	0.0285 (8)	0.0168 (7)	0.0142 (6)	0.0232 (7)
C5	0.0303 (8)	0.0443 (8)	0.0394 (8)	0.0094 (6)	0.0119 (6)	0.0263 (7)
C6	0.0299 (7)	0.0298 (7)	0.0296 (7)	0.0115 (6)	0.0099 (6)	0.0138 (6)
C7	0.0291 (7)	0.0248 (6)	0.0240 (7)	0.0063 (5)	0.0094 (5)	0.0102 (5)
C8	0.0238 (7)	0.0240 (6)	0.0219 (6)	0.0079 (5)	0.0073 (5)	0.0108 (5)
C9	0.0243 (6)	0.0216 (6)	0.0188 (6)	0.0083 (5)	0.0072 (5)	0.0092 (5)
C10	0.0229 (6)	0.0225 (6)	0.0210 (6)	0.0072 (5)	0.0066 (5)	0.0081 (5)
C11	0.0286 (7)	0.0282 (6)	0.0230 (7)	0.0092 (5)	0.0049 (5)	0.0106 (5)
C12	0.0309 (8)	0.0338 (7)	0.0318 (8)	0.0121 (6)	0.0013 (6)	0.0085 (6)
C13	0.0264 (8)	0.0372 (8)	0.0450 (9)	0.0078 (6)	0.0061 (7)	0.0093 (7)
C14	0.0276 (8)	0.0352 (8)	0.0442 (9)	0.0035 (6)	0.0120 (7)	0.0145 (7)
C15	0.0285 (7)	0.0301 (7)	0.0295 (7)	0.0067 (6)	0.0111 (6)	0.0140 (6)
C16	0.0265 (7)	0.0262 (6)	0.0219 (6)	0.0114 (5)	0.0088 (5)	0.0123 (5)
C17	0.0295 (8)	0.0356 (7)	0.0295 (7)	0.0114 (6)	0.0042 (6)	0.0136 (6)
C18	0.0355 (9)	0.0569 (10)	0.0353 (8)	0.0190 (8)	0.0016 (7)	0.0228 (7)
C19	0.0482 (10)	0.0627 (11)	0.0443 (9)	0.0370 (9)	0.0182 (8)	0.0340 (8)
C20	0.0511 (10)	0.0379 (8)	0.0391 (8)	0.0276 (7)	0.0211 (7)	0.0244 (7)
C21	0.0378 (8)	0.0276 (7)	0.0282 (7)	0.0158 (6)	0.0107 (6)	0.0142 (5)
C22	0.0338 (8)	0.0268 (7)	0.0280 (7)	0.0129 (6)	0.0142 (6)	0.0067 (5)
C23	0.0303 (7)	0.0246 (6)	0.0224 (7)	0.0103 (5)	0.0080 (5)	0.0068 (5)
C24	0.0475 (9)	0.0219 (6)	0.0245 (7)	0.0128 (6)	0.0112 (6)	0.0096 (5)
C25	0.0369 (9)	0.0403 (8)	0.0265 (7)	0.0126 (7)	0.0036 (6)	0.0054 (6)
C26	0.0498 (10)	0.0284 (7)	0.0339 (8)	0.0173 (7)	0.0133 (7)	0.0064 (6)
B1	0.0235 (7)	0.0238 (7)	0.0179 (7)	0.0080 (5)	0.0047 (5)	0.0094 (5)
O1	0.0400 (6)	0.0207 (4)	0.0199 (5)	0.0090 (4)	0.0112 (4)	0.0082 (4)
O2	0.0363 (6)	0.0229 (4)	0.0255 (5)	0.0100 (4)	0.0153 (4)	0.0083 (4)

### Geometric parameters (Å, º)

**Table:**

C1—C6	1.3902 (19)	C16—C17	1.531 (2)
C1—C2	1.3983 (18)	C16—C21	1.5316 (17)
C1—C7	1.4777 (17)	C16—H16	1.0000
C2—C3	1.388 (2)	C17—C18	1.532 (2)
C2—H2	0.9500	C17—H17A	0.9900
C3—C4	1.379 (2)	C17—H17B	0.9900
C3—H3	0.9500	C18—C19	1.528 (2)
C4—C5	1.381 (2)	C18—H18A	0.9900
C4—H4	0.9500	C18—H18B	0.9900
C5—C6	1.3879 (19)	C19—C20	1.524 (3)
C5—H5	0.9500	C19—H19A	0.9900
C6—H6	0.9500	C19—H19B	0.9900
C7—C8	1.3264 (18)	C20—C21	1.5338 (19)
C7—H7	0.9500	C20—H20A	0.9900
C8—C9	1.5251 (16)	C20—H20B	0.9900
C8—H8	0.9500	C21—H21A	0.9900
C9—C16	1.5672 (17)	C21—H21B	0.9900
C9—C10	1.5717 (18)	C22—O2	1.4424 (15)
C9—B1	1.6034 (18)	C22—C23	1.5238 (19)
C10—C11	1.5369 (18)	C22—H22A	0.9900
C10—C15	1.5386 (17)	C22—H22B	0.9900
C10—H10	1.0000	C23—C24	1.5227 (18)
C11—C12	1.525 (2)	C23—C25	1.523 (2)
C11—H11A	0.9900	C23—C26	1.5285 (18)
C11—H11B	0.9900	C24—O1	1.4408 (15)
C12—C13	1.523 (2)	C24—H24A	0.9900
C12—H12A	0.9900	C24—H24B	0.9900
C12—H12B	0.9900	C25—H25A	0.9800
C13—C14	1.524 (2)	C25—H25B	0.9800
C13—H13A	0.9900	C25—H25C	0.9800
C13—H13B	0.9900	C26—H26A	0.9800
C14—C15	1.528 (2)	C26—H26B	0.9800
C14—H14A	0.9900	C26—H26C	0.9800
C14—H14B	0.9900	B1—O1	1.3565 (17)
C15—H15A	0.9900	B1—O2	1.3682 (16)
C15—H15B	0.9900		
			
C6—C1—C2	117.89 (12)	C17—C16—H16	106.8
C6—C1—C7	122.74 (12)	C21—C16—H16	106.8
C2—C1—C7	119.37 (12)	C9—C16—H16	106.8
C3—C2—C1	120.89 (14)	C18—C17—C16	111.14 (12)
C3—C2—H2	119.6	C18—C17—H17A	109.4
C1—C2—H2	119.6	C16—C17—H17A	109.4
C4—C3—C2	120.37 (14)	C18—C17—H17B	109.4
C4—C3—H3	119.8	C16—C17—H17B	109.4
C2—C3—H3	119.8	H17A—C17—H17B	108.0
C3—C4—C5	119.46 (13)	C19—C18—C17	111.29 (13)
C3—C4—H4	120.3	C19—C18—H18A	109.4
C5—C4—H4	120.3	C17—C18—H18A	109.4
C4—C5—C6	120.36 (14)	C19—C18—H18B	109.4
C4—C5—H5	119.8	C17—C18—H18B	109.4
C6—C5—H5	119.8	H18A—C18—H18B	108.0
C5—C6—C1	121.03 (13)	C20—C19—C18	111.51 (12)
C5—C6—H6	119.5	C20—C19—H19A	109.3
C1—C6—H6	119.5	C18—C19—H19A	109.3
C8—C7—C1	125.85 (12)	C20—C19—H19B	109.3
C8—C7—H7	117.1	C18—C19—H19B	109.3
C1—C7—H7	117.1	H19A—C19—H19B	108.0
C7—C8—C9	127.42 (12)	C19—C20—C21	111.18 (13)
C7—C8—H8	116.3	C19—C20—H20A	109.4
C9—C8—H8	116.3	C21—C20—H20A	109.4
C8—C9—C16	109.58 (10)	C19—C20—H20B	109.4
C8—C9—C10	110.43 (10)	C21—C20—H20B	109.4
C16—C9—C10	111.93 (10)	H20A—C20—H20B	108.0
C8—C9—B1	109.36 (10)	C16—C21—C20	110.77 (12)
C16—C9—B1	108.39 (10)	C16—C21—H21A	109.5
C10—C9—B1	107.06 (10)	C20—C21—H21A	109.5
C11—C10—C15	109.13 (11)	C16—C21—H21B	109.5
C11—C10—C9	110.54 (10)	C20—C21—H21B	109.5
C15—C10—C9	115.16 (11)	H21A—C21—H21B	108.1
C11—C10—H10	107.2	O2—C22—C23	112.27 (10)
C15—C10—H10	107.2	O2—C22—H22A	109.2
C9—C10—H10	107.2	C23—C22—H22A	109.2
C12—C11—C10	112.66 (11)	O2—C22—H22B	109.2
C12—C11—H11A	109.1	C23—C22—H22B	109.2
C10—C11—H11A	109.1	H22A—C22—H22B	107.9
C12—C11—H11B	109.1	C24—C23—C25	111.00 (12)
C10—C11—H11B	109.1	C24—C23—C22	107.48 (11)
H11A—C11—H11B	107.8	C25—C23—C22	110.22 (12)
C13—C12—C11	111.29 (12)	C24—C23—C26	108.90 (12)
C13—C12—H12A	109.4	C25—C23—C26	110.06 (12)
C11—C12—H12A	109.4	C22—C23—C26	109.12 (11)
C13—C12—H12B	109.4	O1—C24—C23	112.89 (11)
C11—C12—H12B	109.4	O1—C24—H24A	109.0
H12A—C12—H12B	108.0	C23—C24—H24A	109.0
C12—C13—C14	110.08 (12)	O1—C24—H24B	109.0
C12—C13—H13A	109.6	C23—C24—H24B	109.0
C14—C13—H13A	109.6	H24A—C24—H24B	107.8
C12—C13—H13B	109.6	C23—C25—H25A	109.5
C14—C13—H13B	109.6	C23—C25—H25B	109.5
H13A—C13—H13B	108.2	H25A—C25—H25B	109.5
C13—C14—C15	111.34 (12)	C23—C25—H25C	109.5
C13—C14—H14A	109.4	H25A—C25—H25C	109.5
C15—C14—H14A	109.4	H25B—C25—H25C	109.5
C13—C14—H14B	109.4	C23—C26—H26A	109.5
C15—C14—H14B	109.4	C23—C26—H26B	109.5
H14A—C14—H14B	108.0	H26A—C26—H26B	109.5
C14—C15—C10	111.18 (12)	C23—C26—H26C	109.5
C14—C15—H15A	109.4	H26A—C26—H26C	109.5
C10—C15—H15A	109.4	H26B—C26—H26C	109.5
C14—C15—H15B	109.4	O1—B1—O2	122.35 (11)
C10—C15—H15B	109.4	O1—B1—C9	119.72 (11)
H15A—C15—H15B	108.0	O2—B1—C9	117.93 (11)
C17—C16—C21	108.63 (11)	B1—O1—C24	120.49 (10)
C17—C16—C9	112.83 (11)	B1—O2—C22	119.36 (10)
C21—C16—C9	114.57 (11)		

### Hydrogen-bond geometry (Å, º)

**Table:**

*D*—H···*A*	*D*—H	H···*A*	*D*···*A*	*D*—H···*A*
C22—H22*B*···O2^i^	0.99	2.69	3.5773 (18)	150

Symmetry code: (i) −*x*+1, −*y*, −*z*.
